# Two Draft Genome Sequences of *Chromobacterium violaceum* Isolates from the Rio Negro

**DOI:** 10.1128/genomeA.01348-17

**Published:** 2018-01-04

**Authors:** Auricélia Matos da Gama, Luiz Gustavo de Almeida, Tetsuo Yamane, Beny Spira

**Affiliations:** a Departamento de Microbiologia, Instituto de Ciências Biomédicas, Universidade de São Paulo, São Paulo, Brazil; b Escola Superior de Ciências da Saúde, Curso de Farmácia, Universidade do Estado do Amazonas, Manuas, Amazonas, Brazil

## Abstract

The draft genome sequences of two *Chromobacterium violaceum* strains isolated from the Rio Negro are reported here. These bacteria carry most genetic systems associated with the production of bioactive compounds, but unlike other *C. violaceum* strains, they lack a dedicated operon for arsenic resistance.

## GENOME ANNOUNCEMENT

The betaproteobacterium *Chromobacterium violaceum*, found in tropical environments around the globe, forms violet colonies on rich solid media due to the production of violacein (1, 2). *C. violaceum* can cause infections in humans ranging from mild diarrhea to death (3, 4). In addition to violacein, *C. violaceum* produces several potential biotechnology products (5). Though not considered oligotrophic, *C. violaceum* thrives in nutrient-scarce environments, such as the Rio Negro.

Water samples were collected from the Rio Negro at the Amazon Basin (Manaus, Brazil) and spread on l-agar plates. Two violet colonies that are prototrophic, ampicillin resistant, and grow under low-phosphate conditions (6) were isolated. The isolates were designated CV1192 and CV1197.

Genomic DNA was extracted using the Wizard genomic DNA purification kit (Promega) and quantified in a Qubit fluorimeter (Life Technologies). Genome sequencing was performed by the MicrobesNG facility (Birmingham, UK). The genomic DNA library was prepared using the Nextera XT library prep kit (Illumina). Libraries were sequenced on an Illumina HiSeq platform using a 250-bp paired-end protocol. Reads were adapter trimmed using Trimmomatic 0.30, with a sliding window quality cutoff of Q15 (7), and *de novo* genome assembly was carried out with SPAdes (version 3.7) (8). General genome features and annotation were obtained using the Geneious 10.3 and Prokka (9) softwares, with the genome of *C. violaceum* ATCC 12472 (GenBank accession number NC_005085) as a reference.

Each genome comprised 58 contigs, with 50 contigs larger than 1,000 bp. The genome lengths of strains CV1192 and CV1197 are, respectively, 4,386,139 bp (64.9% G+C content) and 4,388,579 bp (65.1% G+C content). Both contain 4,405 coding DNA sequences (CDSs). Of these, 61.08% were assigned a putative function, and the remainder were considered hypothetical. Twenty-five rRNAs and 98 tRNA sequences were annotated. One intact prophage region was detected in both sequences using the PHASTER algorithm (10).

The genomes of CV1192 and CV1197 were, respectively, 92.3% and 92.4% identical to that of *C. violaceum* ATCC 12472. The genomes of both strains lack 61 open reading frames (ORFs) that are present in the ATCC 12472 strain. Of significance, an Rhs-like protein (CV_1238) that is involved in bacteriocin synthesis, a phenazine biosynthesis protein (CV_2663), and the *arsRCB* operon (CV_2438, CV_2439, and CV_2440) are missing in both isolates. Most other missing ORFs code for structural phage proteins or secretion systems. All other genetic elements associated with the production of antibiotics and bioactive secondary metabolites are present in both genomes.

Unlike CV1192 and CV1197, the ATCC 12472 and other *C. violaceum* strains carry a functional *arsRCB* operon, which is involved in arsenic detoxification (11). The concentration of As in the waters of the Rio Negro is approximately 0.05 mg/m^3^ (0.36 nM), being the lowest As concentration among all Amazon rivers (12). The loss of the *arsRCB* operon in CV1192 and CV1197 appears to be a striking example of negative (purifying) selection. Likewise, *Chromobacterium amazonense*, another bacterium isolated from the Rio Negro, lacks an *arsRCB* operon.

### Accession number(s).

The whole-genome shotgun projects have been deposited at DDBJ/EMBL/GenBank under the accession numbers CP024028 (strain CV1192) and CP024029 (strain CV1197).
